# No evidence that vitamin D is able to prevent or affect the severity of COVID-19 in individuals with European ancestry: a Mendelian randomisation study of open data

**DOI:** 10.1136/bmjnph-2020-000151

**Published:** 2021-01-07

**Authors:** Hasnat A Amin, Fotios Drenos

**Affiliations:** 1 Department of Life Sciences, Brunel University London, Uxbridge, UK; 2 Institute of Cardiovascular Sciences, UCL, London, UK

**Keywords:** infectious disease, nutrient deficiencies, nutritional treatment, pulmonary disease

## Abstract

**Background:**

Upper respiratory tract infections are reportedly more frequent and more severe in individuals with lower vitamin D levels. Based on these findings, it has been suggested that vitamin D can prevent or reduce the severity of COVID-19.

**Methods:**

We used two-sample Mendelian randomisation (MR) to assess the causal effect of vitamin D levels on SARS-CoV-2 infection risk and COVID-19 severity using publicly available data. We also carried out a genome-wide association analysis (GWA) of vitamin D deficiency in the UK Biobank (UKB) and used these results and two-sample MR to assess the causal effect of vitamin D deficiency on SARS-CoV-2 infection risk and COVID-19 severity.

**Results:**

We found no evidence that vitamin D levels causally affect the risk of SARS-CoV-2 infection (ln(OR)=0.17 (95% CI −0.22 to 0.57, p=0.39)) nor did we find evidence that vitamin D levels causally affect COVID-19 severity (ln(OR)=0.36 (95% CI −0.89 to 1.61, p=0.57)). Based on our GWA analysis, we found that 17 independent variants are associated with vitamin D deficiency in the UKB. Using these variants as instruments for our two-sample MR analyses, we found no evidence that vitamin D deficiency causally affects the risk of SARS-CoV-2 infection (ln(OR)=−0.04 (95% CI −0.1 to 0.03, p=0.25)) nor did we find evidence that vitamin D deficiency causally affects COVID-19 severity (ln(OR)=−0.24 (95% CI −0.55 to 0.08, p=0.14)).

**Conclusions:**

In conclusion, we found no evidence that vitamin D is protective against SARS-CoV-2 infection or COVID-19 severity. Our data support the recent statement by the National Institute for Health and Care Excellence that the use of vitamin D supplementation to mitigate COVID-19 is not supported by the available data.

**What this paper adds**

- Uncertainty remains over the use of Vitamin D for the prevention of COVID-19 and the moderation of its symptoms.
- Genetic predisposition for higher levels of vitamin D and for lower chance of vitamin D insufficiency do not have evidence of association with infection from SARS-CoV-2 or severity of COVID-19 following infection.
- Our work supports the current NICE statement that, based on the available evidence, vitamin D should not be considered as protective of infection from SARS-CoV-2 or a way to mitigate its severity.

## Introduction

Vitamin D has lately been the focus of very intense scientific interest, with more than 4500 manuscripts published per year since 2015. Although vitamin D is commonly discussed in terms of bone health and calcium and phosphate homeostasis, evidence has started to emerge that it may also be involved in cancer, the cardiovascular system and inflammation.^1^ With the onset of the COVID-19 pandemic, these findings, in combination with previous reports of vitamin D playing a role in upper respiratory tract (URT) infections and their severity, have resulted in further interest in vitamin D and the potential use of vitamin D supplements to mitigate the spread and severity of COVID-19.

We obtain vitamin D either through our diet, with certain foods such as oily fish and egg yolks being good sources, or through our exposure to ultraviolet B radiation from the sun.^2^ Vitamin D, whether generated or consumed, is biologically inactive and undergoes a complex metabolic process: it is first converted to 25-hydroxyvitamin D (25(OH)D) in the liver and then this is converted in the kidney to 1,25-hydroxyvitamin D, which is the active metabolite.^2^ The commonly assessed vitamin D levels refer to the 25(OH)D metabolite, which is the main circulating form in the body.^2^ Vitamin D deficiency, commonly defined as levels lower than 25 nmol/L, is a common problem both in developed^3^ and in developing countries.^4^ Although it is usually thought to be a problem in countries located at higher latitudes and with darker days,^5^ a high prevalence of vitamin D deficiency is also observed in countries close to the equator.^6^ According to guidance from the National Institute for Health and Care Excellence (NICE), those with low vitamin D levels should be treated with high-dose supplementation for a short period, followed by a lower maintenance dose,^7^ and all adults are advised to take a daily supplement containing 10 mg per day throughout the year to prevent vitamin D deficiency.

URT infections are reportedly more frequent and more severe in individuals with lower vitamin D levels. Although these infections are more common during seasons with darker days, when vitamin D levels are lower, vitamin D has also been correlated with better pulmonary function in young adults^8^ and with lower reporting of coughs and colds;^9^ however, these studies cannot provide evidence of causation. A meta-analysis of randomised controlled trials of vitamin D supplementation for acute respiratory tract infections found a protective effect of vitamin D,^10^ but the study has been criticised for the way the approach was used.^11^ Current evidence for the use of vitamin D supplementation against COVID-19 mainly relies on assumptions based on reports of lower vitamin D being associated with a higher risk of URT infections (please see the review by Lanham-New *et al*).^12^ A handful of studies have used a data-based approach,^13–17^ but these efforts rely on correlations between vitamin D and COVID-19, which are liable to be affected by unobserved or inadequately controlled confounding factors.

Although we will need well-powered and carefully executed randomised trials and a subsequent meta-analysis of the different studies to provide an accurate estimate of the effect of vitamin D on COVID-19 prevention and severity, we can anticipate the results of such studies by comparing individuals who are genetically predisposed to lower vitamin D levels with those who are not, based on the Mendelian randomisation (MR) paradigm. In a randomised controlled trial, we would minimise the effect of confounding factors by randomly assigning participants to a treatment group receiving vitamin D supplements or to a control group receiving a placebo and thus estimate the true effect of the intervention. In the natural experiment of MR, genetic variants predisposing the individual to higher levels of vitamin D are assigned randomly at conception, based on the genetic polymorphisms of their parents, in relation to other possible confounding traits. As genetic polymorphisms remain constant throughout life and the individual does not change their vitamin D intake according to their genotype, the use of this information can provide indirect evidence of causality.^18^ Here, using data from genome-wide association (GWA) studies for vitamin D levels, vitamin D deficiency and COVID-19 incidence and severity, we test whether genetically increased vitamin D levels are associated with SARS-CoV-2 infection risk and COVID-19 severity.

## Methods

### Population and study design

We predominately used previously published and freely available data for this study. The study by Jiang *et al* 19 is a meta-analysis of GWAs of vitamin D levels carried out using participants of European descent. The COVID-19 Host Genetics Initiative^20^ uses data from multiple cohort studies,^21–26^ including the UK Biobank (UKB).

The UKB individual-level data were also used following permission to use data already available for COVID-19-related research. UKB is a large prospective cohort study that recruited >500 000 UK residents between 2006 and 2010. The 22 UKB assessment centres, located throughout England, Wales and Scotland, collected baseline data from the participants in the form of questionnaires, physical and cognitive tests, and blood and urine samples.^27^ The age range of the participants at the time of enrolment in the study was between 40 and 69 years of age, with a mean age of 56.5 years. Men represent 45.6% of the sample. The use of the data for this project was approved by the UKB (application 44566).

### Genotyping

In the UKB, 488 377 individuals had been genotyped for up to 812 428 variants using DNA extracted from blood samples on either the UKB Axiom array (438 427 participants) or the UK BiLEVE Axiom array (49 950 participants). Variants that did not pass standard quality control checks were excluded.^28^ These included tests for the presence of batch effects, plate effects, sex effects and array effects, as well as any departures from Hardy-Weinberg Equilibrium using a p value threshold of 10^−12^. Variants with a minor allele frequency of <0.01 were also excluded.

Sample genotyping quality control metrics were provided by UKB.^28^ Samples were excluded from the analysis if they were outliers for missingness and/or PC-corrected heterozygosity and/or if they had any sex chromosome aneuploidies, as well as if the genetically inferred sex differed from the reported sex. Samples which did not have a genetically determined white British ancestry were also excluded. A list of related individuals was also provided by UKB and one individual from each related pair was excluded at random.

Genetic data from studies used by the COVID-19 Host Genetics Initiative underwent quality control and imputation using the protocol described by Lam *et al.* 29

### Phenotypes

We used summary statistics from phenotypes B1 and C1 by the COVID-19 Host Genetics Initiative (September 2020 release). Phenotype B1 is a measure of COVID-19 severity and only included individuals who were positive for SARS-CoV-2 infection based on an RNA-based and/or a serology-based test: individuals who were hospitalised due to coronavirus-related symptoms were coded as cases; and individuals who were not hospitalised for 21 days or more after their positive SARS-CoV-2 test were coded as controls. Phenotype C1 is a measure of susceptibility to SARS-CoV-2 infection: individuals were coded as cases if they were diagnosed with COVID-19 by a doctor or if they were positive for SARS-CoV-2 infection based on an RNA-based and/or a serology-based test or if they self-reported being positive for COVID-19; and individuals were coded as controls if they tested negative (for all tests if multiple tests were performed) for SARS-CoV-2 infection based on an RNA-based and/or a serology-based test or if they self-reported being negative for COVID-19. Please see https://www.covid19hg.org/about/ for more details.

Vitamin D deficiency: individuals whose vitamin D levels (UKB field 30890) were <25 nmol/L were coded as cases; and individuals whose vitamin D levels were ≥50 nmol/L were coded as controls. COVID-19 test results^30^ from the UKB were made available through linkage to national health records. Obesity: individuals whose body mass index (BMI) was ≥18.5 kg/m^2^ and <25 kg/m^2^ (UKB field 21001) were considered as having a normal weight; and individuals whose BMI was ≥30 kg/m^2^ were considered as being obese. Season: individuals who attended the assessment centres during December, January or February (UKB field 55) were considered as winter samples; and individuals who attended during June, July or August were considered as summer samples.

### Statistical analyses

We used R V.4.0.2^31^ to carry out analyses and generate plots, unless stated otherwise. We used PLINK V.1.9^32^ to carry out genetic association analyses using UKB data and to generate the genetic risk score for vitamin D deficiency.

**Figure 1:**
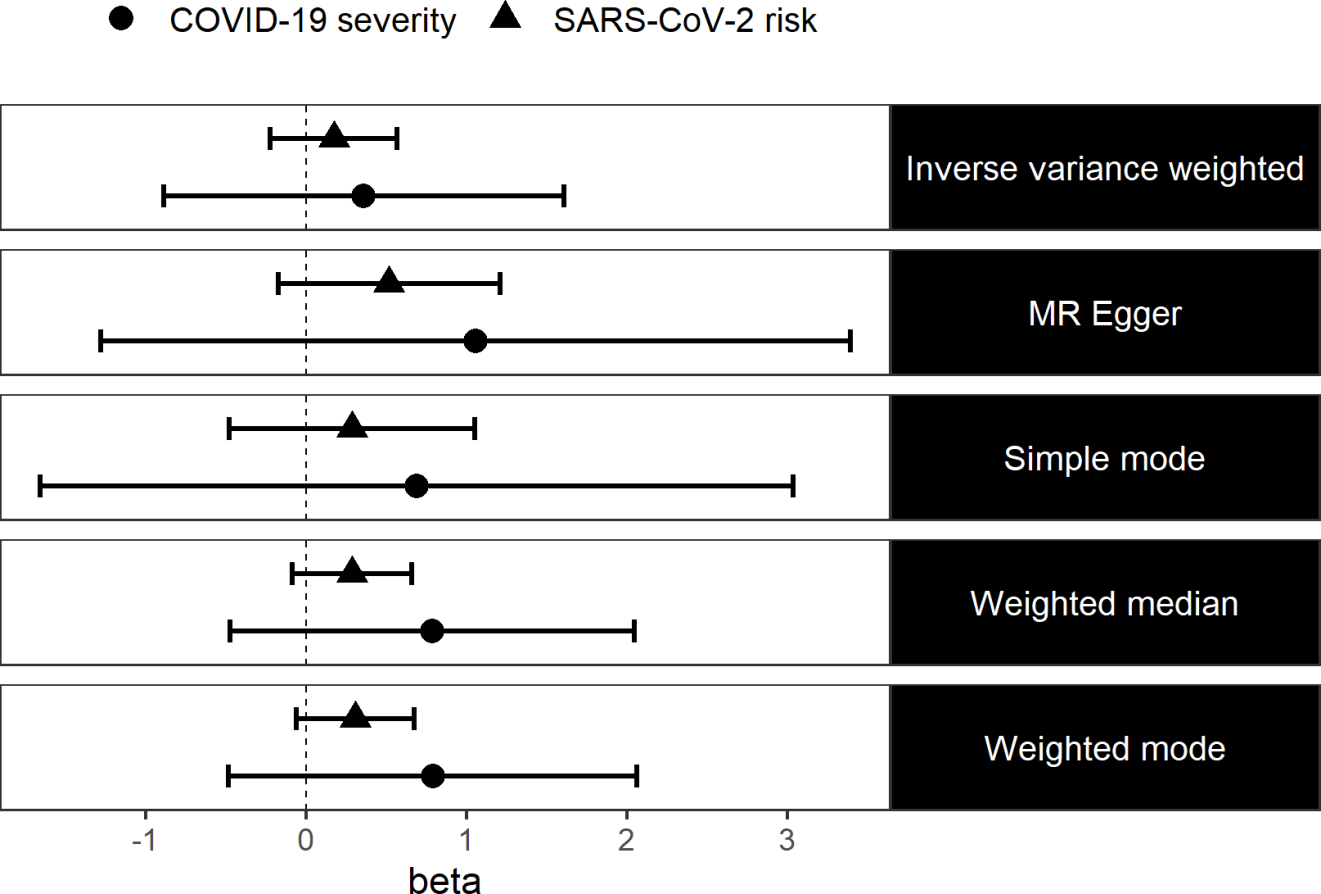
Log ORs (beta) and 95% CIs from a two-sample MR analysis of the effect of vitamin D levels on SARS-CoV-2 risk and COVID-19 severity. MR, Mendelian randomisation.

**Figure 2:**
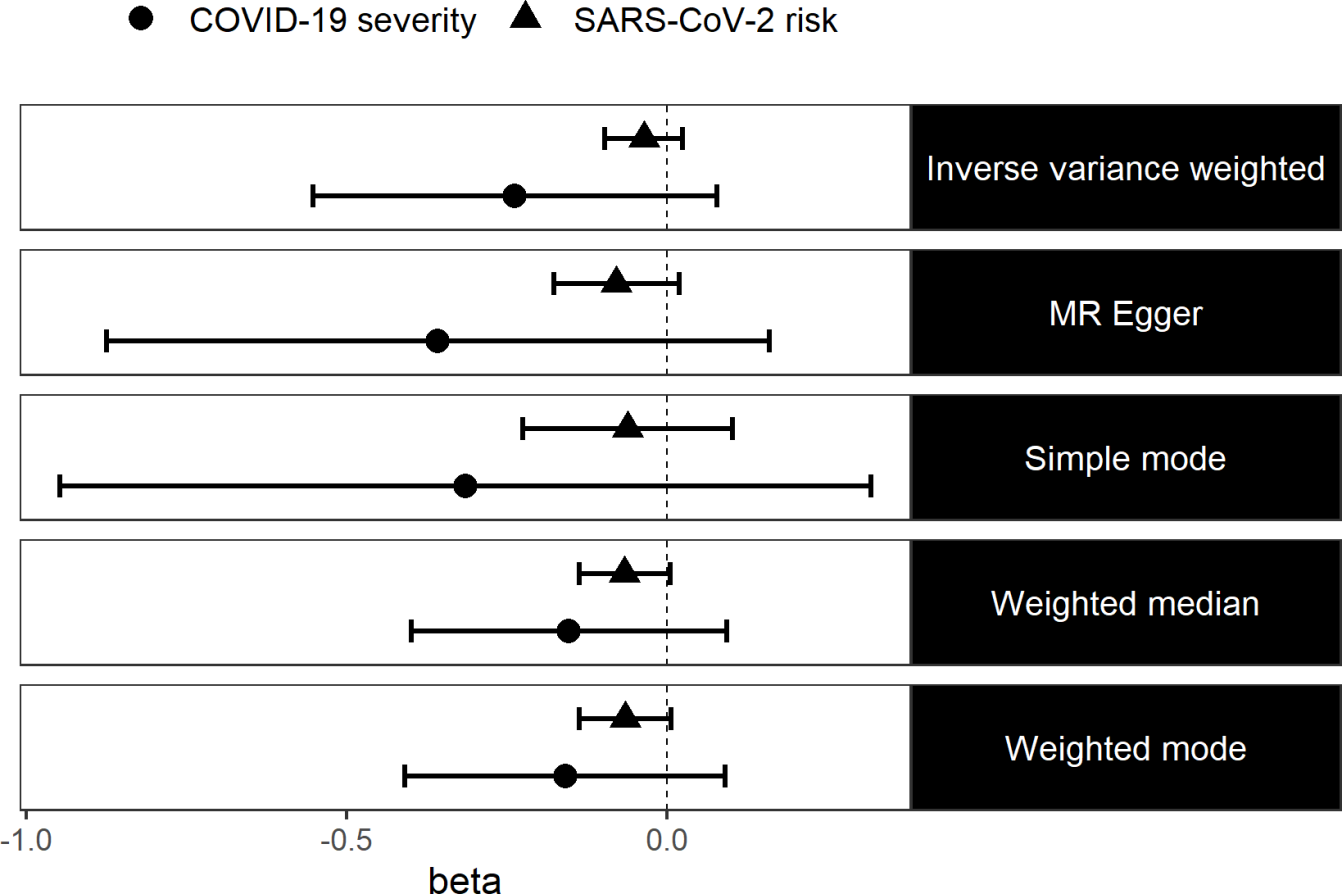
Log ORs (beta) and 95% CIs from a two-sample MR analysis of the effect of vitamin D deficiency on SARS-CoV-2 risk and COVID-19 severity. MR, Mendelian randomisation.

Welch’s two-sample t-test was used to assess the differences in the distribution of vitamin D levels in the following categories: obese versus normal weight; summer samples versus winter samples; and the bottom and top quartiles of the vitamin D levels genetic risk score generated using variants from Jiang *et al* 19 (online supplemental table 1B and figure 1). Fisher’s exact test was used to assess the differences in the prevalence of vitamin D deficiency in the three aforementioned categories, except that the vitamin D deficiency genetic risk score used for this analysis was generated using the 17 variants associated with vitamin D deficiency in the UKB (see online supplemental table 1A and figure 2).

10.1136/bmjnph-2020-000151.supp1 Supplementary data

To assess the causal effect of vitamin D levels on SARS-CoV-2 infection risk and COVID-19 severity, we used outcome summary statistics from the COVID-19 Host Genetics Initiative^20^ (see the Phenotypes section) and exposure summary statistics from Jiang *et al* 19 (see the Population and study design section) to carry out two-sample MR using the TwoSampleMR R package.^33^ Note: rs3755967, rs12785878 and rs8018720 were not available in the summary statistics for phenotypes B1 and C1, so we used rs17467825, rs3794060 and rs8022510, respectively, as proxies (R^2^ >0.99). The causal estimates (beta) are expressed as ln(OR) per ln(nM), where OR=Odds ratio; and nM=concentration of vitamin D in nmol/L.

For each genetic variant that is associated with the exposure of interest (eg, vitamin D), the causal effect of the exposure of interest on the outcome of interest (eg, SARS-CoV-2 risk or COVID-19 severity) can be estimated by calculating the Wald ratio, which is the effect of the variant on the outcome divided by the effect of the variant on the exposure. If there are multiple independently inherited variants associated with the exposure, as in this case, the inverse variance weighted MR (IVW-MR) method is used to provide an overall estimate of the causal effect by calculating a weighted average of the Wald ratios. However, in the presence of pleiotropy (ie, a genetic variant is associated with the outcome through a pathway that does not include the exposure of interest), the estimate from the IVW-MR method may be biased. The MR Egger method models this possible violation of the assumption through the intercept of a linear model between the effect of the instruments on the exposure and outcome. In the absence of pleiotropy, the value of this intercept does not differ from zero. However, if the pleiotropic effects of the variants are related to their effects on the exposure (ie, a violation of the INSIDE assumption), the MR Egger and IVW-MR methods are both susceptible to bias. In this case, the weighted median, simple mode and weighted mode MR methods are used. These methods use the median or mode of the Wald ratios to provide robust estimates in cases where some of the genetic instruments violate the pleiotropy assumption.^34^

In order to use two-sample MR to estimate the causal effect of vitamin D deficiency on the aforementioned outcomes, we needed to obtain new instruments for vitamin D deficiency. We therefore carried out a GWA analysis in the UKB using PLINK V.1.9,^32^ adjusted for the first four principal components for the genetic variability of the genome, age at baseline, sex and the genotyping array used. The associated genes for each variant were obtained from National Center for Biotechnology Information Single Nucleotide Polymorphism (NCBI SNP). As the COVID-19 Host Genetics Initiative uses data from the UKB, there is a possibility that the estimates from two-sample MR analyses may be biased; Burgess *et al* 35 suggest that this bias can be minimised by generating exposure summary statistics using control samples only, so we excluded individuals who had been tested for COVID-19 from our GWA analysis. The summary statistics for vitamin D deficiency were then filtered using a p value threshold of 5×10^−8^ and clumped using the ‘clump_data’ function, which finds the variant with the smallest p value, removes any variants that are in linkage disequilibrium (R^2^ >0.001) and repeats this process until there are no variants remaining. It is possible that the effects of the genetic variants associated with vitamin D deficiency may vary by season, so we repeated the genetic association analyses using winter samples only and used the effect sizes from these to carry out two-sample MR to test the sensitivity of our results to this possibility.

## Results

For SARS-CoV-2 infection susceptibility (phenotype C1), summary statistics from 11 181 cases and 116 456 controls were available as of 30 September 2020. For COVID-19 severity (phenotype B1), summary statistics from 1389 cases and 5879 controls were available as of 30 September 2020. For the vitamin D deficiency phenotype in the UKB, there were 35 079 cases and 140 898 controls. Please see the Methods section for the phenotype definitions. The genetic risk score generated using the six variants from Jiang *et al* 19 (online supplemental table 1B) explained 2.518% of the variance in vitamin D concentration and the genetic risk score generated using the newly identified 17 variants associated with vitamin D deficiency (online supplemental table 1A) explained 2.108% of the variance in vitamin D deficiency (note: the latter is an approximation based on McFadden’s R^2^). Table 1 shows the distribution of vitamin D levels and the prevalence of vitamin D deficiency by genetic risk score category (1st quartile vs 4th quartile), together with the changes associated with known factors affecting vitamin concentration, such as obesity and season.

**Table 1:** Distribution of vitamin D levels and prevalence of vitamin D deficiency by obesity status, season and the genetic risk scores in the UK Biobank

	Vitamin D (SD), nmol/L		Vitamin D deficiency, %	
Gene score		8.4 (8.2 to 8.6)*		2.5 (2.4 to 2.6)†
1st quartile	45.6 (19.0)‡		14§	
4th quartile	54.0 (22.5)‡		29§	
Obesity		−8.7 (−8.9 to −8.5)*		2.5 (2.4 to 2.6)†
Normal BMI	53.0 (21.9)		16	
BMI ≥30 kg/m^2^	44.3 (19.2)		32	
Season		17.1 (17.3 to 16.9)*		17.1 (16.3 to 18.0)†¶
Winter	41.4 (19.1)		42	
Summer	58.6 (19.5)		4	

*Mean difference (95% CIs) from Welch’s two-sample t-test.

†ORs (95% CIs) from Fisher’s exact test.

‡A higher vitamin D level score represents a genetic predisposition to higher vitamin D levels.

§A higher vitamin D deficiency score represents a greater risk for vitamin D deficiency.

¶OR is in the direction of winter.

BMI, body mass index.

We estimated the causal effect of vitamin D levels on the risk of SARS-CoV-2 infection and severe COVID-19 using two-sample MR. We found no evidence in the existing data that vitamin D levels causally affect the risk of SARS-CoV-2 infection (IVW: ln(OR)=0.17 (95% CI −0.22 to 0.57, p=0.39)) nor did we find evidence that vitamin D levels causally affect COVID-19 severity (IVW: ln(OR)=0.36 (95% CI −0.89 to 1.61, p=0.57)). We also used four other more robust MR methods and we still did not find any evidence in the existing data to suggest that vitamin D levels causally affect SARS-CoV-2 risk or COVID-19 severity (figure 1). Testing for the presence of pleiotropy for our genetic instruments using the MR Egger method suggests that our estimates are not biased due to pleiotropy (online supplemental table 2).

It is possible that simply having vitamin D levels that are lower, but still within the optimal range, may not affect SARS-CoV-2 risk nor COVID-19 severity. We therefore performed a GWA analysis of vitamin D deficiency in the UKB, found that 17 independent variants were associated with this phenotype (online supplemental table 1A) and used these variants in a two-sample MR analysis to estimate the causal effect of vitamin D deficiency on the risk of SARS-CoV-2 infection and COVID-19 severity. We found no evidence that vitamin D deficiency causally affects the risk of SARS-CoV-2 infection (IVW: ln(OR)=−0.04 (95% CI −0.1 to 0.03, p=0.25)) nor did we find evidence that vitamin D deficiency causally affects COVID-19 severity (IVW: ln(OR)=−0.24 (95% CI −0.55 to 0.08, p=0.14)). We also used four other robust MR methods and we still did not find any evidence to suggest that vitamin D deficiency causally affects SARS-CoV-2 risk or COVID-19 severity (figure 2). Again, we did not detect any evidence of pleiotropy bias in our results using the MR Egger method (online supplemental table 3). We repeated the two-sample MR using effect sizes from the winter samples only (online supplemental table 4) as a sensitivity analysis, and our results did not differ (online supplemental table 5).

## Discussion

Using previously published results for the genetics of vitamin D levels, UKB individual-level data for the genetics of vitamin D deficiency and the accumulating genetic results for susceptibility and severity of COVID-19, we tested the causal effect of vitamin D levels and deficiency on protection from SARS-CoV-2 infection and COVID-19 severity. We found no evidence that vitamin D is causally related to COVID-19 outcomes and there is no evidence to suggest that current NICE guidance should change to support the use of vitamin D supplementation against COVID-19.

Previously published evidence,^10^ though criticised,^11^ supports the idea that increasing vitamin D levels are protective against acute respiratory tract infections, but these results do not appear to translate in the case of COVID-19. Studies specifically looking at the correlation of vitamin D with COVID-19 and its severity observed an inverse association between them,^13 15–17^ though, in one study,^14^ when a number of possible confounders were adjusted for, the correlation was no longer present. These studies, however, can only provide very limited information on causality and they are sensitive to uncontrolled confounders.

Vitamin D is lower in hospitalised individuals and even more so in those in care homes with limited mobility and exposure to sunlight,^36^ both of which are much more common in the elderly. COVID-19, at least in the UK, has had a disproportional effect on older people and care homes, making it difficult to disentangle the complex relationships between age and disability on one hand and diet and sunlight exposure affecting vitamin D on the other. Our approach uses genetic information to avoid the problem of unobserved confounders, a method that has rapidly gained popularity for the estimation of causal effects based on observational studies.^37^ Our results are based on GWA studies combining data for tens of thousands of individuals from different sources of information to ensure an unbiased estimate and a result that provides the best chance to detect an effect, if present.

However, our work is not without limitations. The most common problem of MR analyses is the presence of pleiotropy. Although this is more likely to cause false positives, rather than false negatives, no evidence for pleiotropy was detected in our analyses. We also used multiple MR models that make slightly different assumptions and provide a more pleiotropy robust result with all of them providing the same conclusion. Vitamin D levels were represented by measures of 25(OH)D which, despite being the most commonly assessed vitamin D metabolite in a clinical setting, does not directly measure the activated form of vitamin D and its measurement and relevance to health are under discussion.^38^ Our results also cannot be used to comment on the relationship between vitamin D and COVID-19 in non-Europeans. Finally, the available data for SARS-CoV-2 infection or severe COVID-19 disease are still limited and a more precise picture will emerge as more information becomes available.

To summarise, using a two-sample MR method, GWA studies of vitamin D and the latest data from tens of thousands of individuals courtesy of the international COVID-19 Host Genetics Initiative, we found no evidence of vitamin D being protective against SARS-CoV-2 infection or severe COVID-19. Our results support the recent statement by NICE that the use of vitamin D supplementation to mitigate COVID-19 is not supported by the available data.
