# Supplementary material for: No evidence that vitamin D is able to prevent or affect the severity of COVID-19 in individuals with European ancestry: a Mendelian randomisation study of open data
Source: BMJ Nutr Prev Health. 2021 Jan 7:bmjnph-2020-000151. doi: 10.1136/bmjnph-2020-000151 (PMC7798425; doi:10.1136/bmjnph-2020-000151)
Supplement: Supplementary file 1 [file bmjnph-2020-000151supp001.pdf]

Supplementary Information S1

Supplementary Table 1

Regression coefficients (beta) and standard errors (se) when (a) regressing vitamin D deficiency on the 17 independent variants associated with vitamin D deficiency in the UK Biobank and (b) when regressing vitamin D concentrations on the 6 independent variants associated with vitamin D concentrations from Jiang *et al.*[19] Gene(s) = nearest/associated genes from NCBI SNP database. Effect allele frequency = eaf. P-value = pval.

	SNP	Gene(s)	effect_allele	eaf	beta	se	pval	other_allele
**a**								
	rs7129781	CYP2R1	C	0.071	0.189	0.016	3.53E-33	T
	rs4944958	NADSYN1	A	0.203	0.259	0.01	8.74E-143	G
	rs964184	ZPR1	G	0.129	0.095	0.012	2.61E-14	C
	rs10859995	HAL	T	0.42	-0.105	0.009	3.01E-33	C
	rs1532085	Intergenic	A	0.383	0.05	0.009	1.15E-08	G
	rs1800588	LIPC	T	0.212	0.066	0.01	1.92E-10	C
	rs55791371	Intergenic	C	0.121	-0.081	0.013	1.27E-09	A
	rs10426201	SULT2A1	G	0.169	-0.106	0.012	8.17E-20	A
	rs3750297	PADI1	A	0.283	0.059	0.009	2.72E-10	G
	rs12123821	LOC112268240	T	0.047	-0.227	0.022	6.25E-26	C
	rs4845491	SMCP	C	0.054	-0.119	0.019	7.46E-10	T
	rs8123293	Intergenic	G	0.113	-0.076	0.014	2.52E-08	A
	rs17217119	Intergenic	G	0.193	0.086	0.011	4.95E-16	A
	rs3755322	UGT1A5<br>UGT1A10<br>UGT1A6<br>UGT1A8<br>UGT1A7<br>UGT1A9	G	0.083	0.104	0.015	5.68E-12	C
	rs6600893	UGT2B7	C	0.452	0.067	0.009	5.49E-15	T
	rs2282679	GC	G	0.274	0.429	0.009	1.00E-200	T
	rs2205262	LINC00536	C	0.429	-0.057	0.009	5.13E-11	A
**b**								
	rs3755967	GC	T	0.28	-0.089	0.002	4.74E–343	C
	rs12785878	NADSYN1/ DHCR7	T	0.75	0.036	0.002	3.80E–62	G
	rs10741657	CYP2R1	A	0.4	0.031	0.002	2.05E–46	G
	rs17216707	CYP24A1	T	0.79	0.026	0.003	8.14E–23	C
	rs10745742	AMDHD1	T	0.4	0.017	0.002	1.88E–14	C
	rs8018720	SEC23A	C	0.82	-0.017	0.003	4.72E–09	G

Amin HA, Drenos F. *bmjnph* 2021;0:1–7. doi: 10.1136/bmjnph-2020-000151

Supplementary Table 2

Log odds ratio (beta), upper (upper) and lower (lower) 95% confidence intervals and MR Egger intercept p-values (p_intercept) from a two-sample MR analysis of the effect of vitamin D levels on SARS-CoV-2 risk and COVID-19 severity.

outcome	method	beta	se	upper	lower	p	p_intercept
COVID-19 severity	MR Egger	1.06	1.19	3.39	-1.28	0.43	0.52
COVID-19 severity	Weighted median	0.79	0.64	2.05	-0.47	0.22	NA
COVID-19 severity	Inverse variance weighted	0.36	0.64	1.61	-0.89	0.57	NA
COVID-19 severity	Simple mode	0.69	1.20	3.04	-1.66	0.59	NA
COVID-19 severity	Weighted mode	0.79	0.65	2.07	-0.48	0.28	NA
SARS-CoV-2 risk	MR Egger	0.52	0.35	1.21	-0.17	0.22	0.31
SARS-CoV-2 risk	Weighted median	0.29	0.19	0.66	-0.09	0.13	NA
SARS-CoV-2 risk	Inverse variance weighted	0.17	0.20	0.57	-0.22	0.39	NA
SARS-CoV-2 risk	Simple mode	0.29	0.39	1.05	-0.48	0.49	NA
SARS-CoV-2 risk	Weighted mode	0.31	0.19	0.67	-0.06	0.16	NA

Amin HA, Drenos F. *bmjnph* 2021;0:1–7. doi: 10.1136/bmjnph-2020-000151

Supplementary Table 3

Log odds ratio (beta), upper (upper) and lower (lower) 95% confidence intervals and MR Egger intercept p-values (p_intercept) from a two-sample MR analysis of the effect of vitamin D deficiency on SARS-CoV-2 risk and COVID-19 severity.

outcome	method	beta	se	upper	lower	p	p_intercept
COVID-19 severity	MR Egger	-0.36	0.26	0.16	-0.87	0.20	0.57
COVID-19 severity	Weighted median	-0.15	0.13	0.09	-0.40	0.22	NA
COVID-19 severity	Inverse variance weighted	-0.24	0.16	0.08	-0.55	0.14	NA
COVID-19 severity	Simple mode	-0.31	0.32	0.32	-0.95	0.35	NA
COVID-19 severity	Weighted mode	-0.16	0.13	0.09	-0.41	0.24	NA
SARS-CoV-2 risk	MR Egger	-0.08	0.05	0.02	-0.18	0.14	0.29
SARS-CoV-2 risk	Weighted median	-0.07	0.04	0.01	-0.14	0.07	NA
SARS-CoV-2 risk	Inverse variance weighted	-0.04	0.03	0.03	-0.10	0.25	NA
SARS-CoV-2 risk	Simple mode	-0.06	0.08	0.10	-0.22	0.48	NA
SARS-CoV-2 risk	Weighted mode	-0.06	0.04	0.01	-0.14	0.10	NA

Amin HA, Drenos F. *bmjnph* 2021;0:1–7. doi: 10.1136/bmjnph-2020-000151

Supplementary Figure 1

Regression coefficients and 95% confidence intervals when regressing vitamin D concentrations on the 6 independent variants associated with vitamin D concentrations from Jiang *et al*.[19]

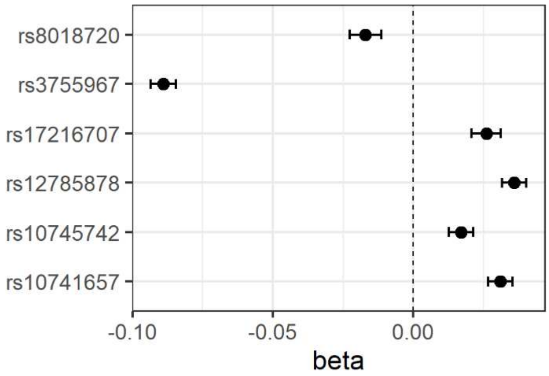

Supplementary Figure 2

Regression coefficients and 95% confidence intervals when regressing vitamin D deficiency on the 17 independent variants associated with vitamin D deficiency in the UK Biobank.

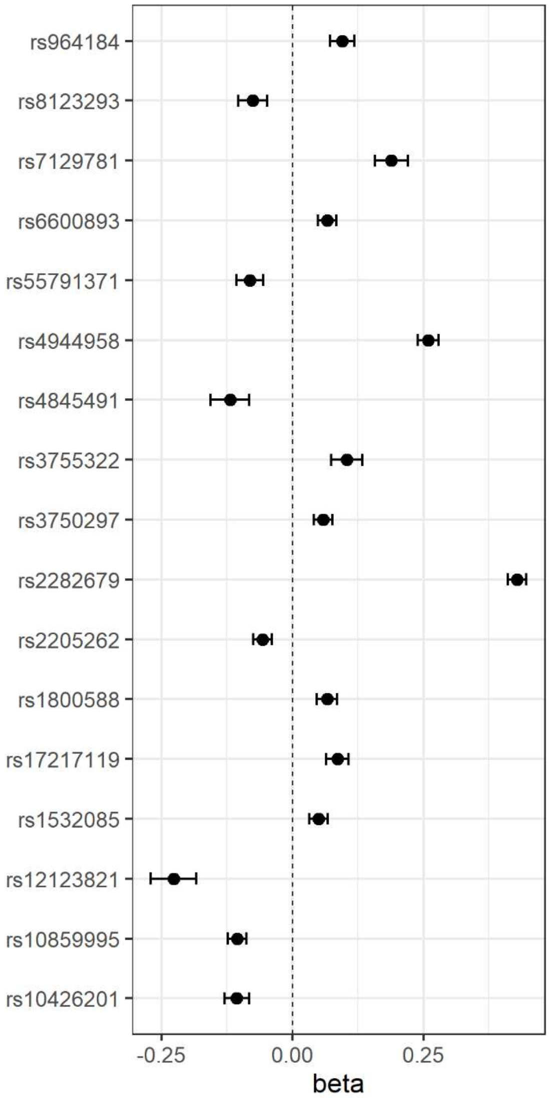

Amin HA, Drenos F. *bmjnph* 2021;0:1–7. doi: 10.1136/bmjnph-2020-000151

Supplementary Table 4

Regression coefficients (beta) and standard errors (se) when regressing vitamin D deficiency on the 17 independent variants associated with vitamin D deficiency in the UK Biobank using winter samples only. Gene(s) = nearest/associated genes from NCBI SNP database. Effect allele frequency = eaf. P-value = pval.

SNP	Gene(s)	effect_allele	eaf	beta	se	pval	other_allele
rs7129781	CYP2R1	C	0.072	0.159	0.032	6.01E-07	T
rs4944958	NADSYN1	A	0.208	0.283	0.020	5.91E-44	G
rs964184	ZPR1	G	0.133	0.075	0.024	2.19E-03	C
rs10859995	HAL	T	0.413	-0.116	0.017	7.47E-12	C
rs1532085	Intergenic	A	0.384	0.043	0.017	1.20E-02	G
rs1800588	LIPC	T	0.215	0.086	0.020	1.81E-05	C
rs55791371	Intergenic	C	0.119	-0.092	0.026	3.51E-04	A
rs10426201	SULT2A1	G	0.170	-0.132	0.022	3.17E-09	A
rs3750297	PADI1	A	0.288	0.069	0.018	1.62E-04	G
rs12123821	LOC112268240	T	0.046	-0.256	0.041	4.88E-10	C
rs4845491	SMCP	C	0.055	-0.120	0.037	1.23E-03	T
rs8123293	Intergenic	G	0.113	-0.092	0.027	5.41E-04	A
rs17217119	Intergenic	G	0.194	0.098	0.021	2.76E-06	A
rs3755322	UGT1A5<br>UGT1A10<br>UGT1A6<br>UGT1A8<br>UGT1A7<br>UGT1A9	G	0.083	0.091	0.030	2.47E-03	C
rs6600893	UGT2B7	C	0.456	0.073	0.017	1.24E-05	T
rs2282679	GC	G	0.284	0.504	0.019	6.81E-162	T
rs2205262	LINC00536	C	0.428	-0.065	0.017	1.03E-04	A

Amin HA, Drenos F. bmjnph 2021;0:1–7. doi: 10.1136/bmjnph-2020-000151

Supplementary Table 5

Log odds ratio (beta), upper (upper) and lower (lower) 95% confidence intervals and MR Egger intercept p-values (p_intercept) from a two-sample MR analysis of the effect of vitamin D deficiency on SARS-CoV-2 risk and COVID-19 severity using winter effect sizes.

outcome	method	beta	se	upper	lower	p	p_intercept
COVID-19 severity	MR Egger	-0.29	0.23	0.15	-0.74	0.22	0.63
COVID-19 severity	Weighted median	-0.13	0.11	0.09	-0.35	0.24	NA
COVID-19 severity	Inverse variance weighted	-0.21	0.14	0.07	-0.49	0.15	NA
COVID-19 severity	Simple mode	-0.28	0.31	0.33	-0.89	0.39	NA
COVID-19 severity	Weighted mode	-0.14	0.10	0.06	-0.33	0.20	NA
SARS-CoV-2 risk	MR Egger	-0.07	0.04	0.01	-0.16	0.11	0.26
SARS-CoV-2 risk	Weighted median	-0.06	0.03	0.01	-0.12	0.07	NA
SARS-CoV-2 risk	Inverse variance weighted	-0.04	0.03	0.02	-0.09	0.20	NA
SARS-CoV-2 risk	Simple mode	-0.06	0.07	0.08	-0.20	0.44	NA
SARS-CoV-2 risk	Weighted mode	-0.06	0.03	0.00	-0.12	0.08	NA

Amin HA, Drenos F. *bmjnph* 2021;0:1–7. doi: 10.1136/bmjnph-2020-000151
